# Immune Checkpoint Inhibitors and Kidney Toxicity: Advances in Diagnosis and Management

**DOI:** 10.1016/j.xkme.2021.08.008

**Published:** 2021-10-08

**Authors:** Harish Seethapathy, Sandra M. Herrmann, Meghan E. Sise

**Affiliations:** 1 Department of Internal Medicine, Division of Nephrology, Massachusetts General Hospital, Boston, MA; 2 Department of Internal Medicine, Division of Nephrology and Hypertension, Mayo Clinic, Rochester, MN

**Keywords:** Immune checkpoint inhibitor, acute kidney injury, chronic kidney disease, renal failure, nephrotoxicity, acute interstitial nephritis

## Abstract

Immune checkpoint inhibitors are now approved for more than 50 indications, and increasing numbers of patients with advanced cancer are receiving immunotherapy. Immune-related adverse events that result from checkpoint inhibitors can affect any organ system. The most common kidney side effect is acute kidney injury, typically caused by acute interstitial nephritis. This review covers the most recent advances in immune checkpoint inhibitor-induced acute kidney injury. The review focuses on the differences between checkpoint inhibitor classes in causing acute kidney injury and differentiating immune checkpoint inhibitor-induced kidney damage from other causes of acute kidney injury. We describe the appropriate use of a kidney biopsy in the diagnosis of acute kidney injury and highlight the need for identification of noninvasive diagnostic and predictive biomarkers of immune checkpoint inhibitor-induced acute kidney injury. In the treatment section, approaches to corticosteroid use and the risks and benefits of rechallenging patients who experience acute kidney injury are debated. We also clarify the long-term adverse effects of immune checkpoint inhibitors on kidney function and the risk of chronic kidney disease in cancer survivors.

## Introduction

Immune checkpoint inhibitors (ICIs) are now considered standard of care in the management of many advanced cancers. Since the approval of the first immune checkpoint inhibitor (ipilimumab) for metastatic melanoma by the US Food and Drug Administration in 2011, 6 additional agents have been approved and their clinical use has expanded to more than 19 distinct cancers.^1^^,^^2^ Harnessing the potency of the immune system and unleashing T cells against cancer tissue has been a breakthrough in cancer treatment. However, widespread T-cell disinhibition also leads to autoimmune side effects, and managing these immune-mediated adverse events is an important part of the clinical care of patients receiving ICIs.^3^ Immune-related adverse events can affect any organ system, with severity ranging from mild to life-threatening. Acute kidney injury (AKI) after ICIs was noted in early case reports with acute interstitial nephritis (AIN) as the most common pattern of injury.^4^, ^5^, ^6^ Guidelines for management of ICI-induced AKI have been created based on expert opinion.^7^, ^8^, ^9^ In recent years, our understanding of ICI-related kidney adverse events has grown substantially. There is a growing body of literature highlighting the different patterns of injury associated with these drugs, the differing manifestations of the ICI classes by virtue of their mechanism of action, and approaches to management. In this review, we discuss recent advances that improve our understanding of the challenges involved in the diagnosis and management of patients who experience kidney dysfunction after ICI treatment.

## Diagnosis

### Distinguishing ICI and non-ICI-induced AKI and risk factors

**Figure 1:**
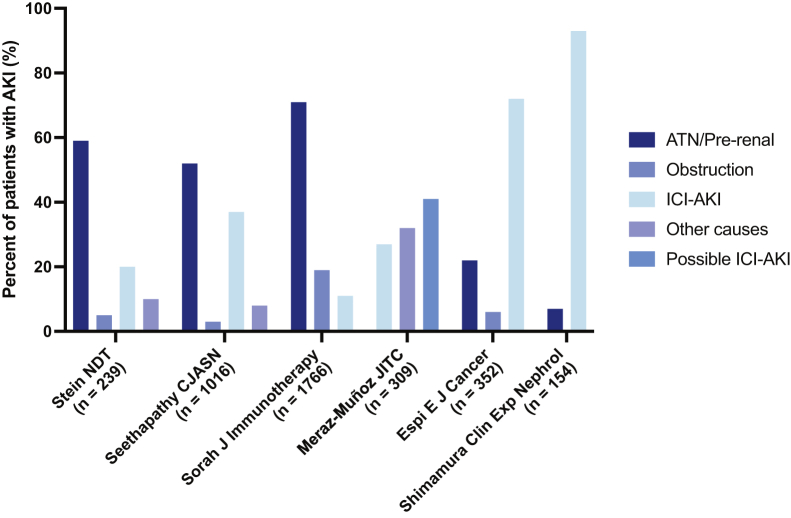
Breakdown of acute kidney injury (AKI) etiologies in single-center cohort studies of patients receiving immune checkpoint inhibitors (ICIs). The etiology of AKI was adjudicated by 2 nephrologists through a manual review of the electronic medical record, with a third available for disagreement.^10^, ^11^, ^12^^,^^16^, ^17^, ^18^ Abbreviation: ATN, acute tubular necrosis.

AKI is common in patients with cancer, and cohort studies suggest that AKI in patients receiving ICIs is more commonly caused by non–ICI-related causes than ICI-induced AKI. Studies from different centers from around the world indicate that the overall incidence of AKI in patients receiving ICIs is approximately 17%, while the incidence of ICI-related AKI is around 2%-5%.^6^^,^^10^, ^11^, ^12^, ^13^, ^14^, ^15^ In cancer patients, AKI is most often caused by prerenal causes such as volume depletion or sepsis; more than 50% of AKI events occur due to hemodynamic insults leading to prerenal or intrinsic kidney injury, though there is variability in single-center reports (Fig 1).^10^, ^11^, ^12^^,^^16^, ^17^, ^18^ Hence, a thorough history and physical examination evaluating for the common causes of AKI are important to avoid an expensive work-up. If there are no obvious hemodynamic insults, clinicians should look for certain risk factors that provide clues to the presence of ICI-induced AKI. Low baseline-estimated glomerular filtration rate and medications that can potentially induce an allergic immune response such as proton pump inhibitors (PPI) are risk factors for ICI-related AKI.^12^^,^^19^^,^^20^ PPIs are an important cause of AIN on their own and have been very commonly reported as concomitant medications in patients who develop ICI-related AIN. In a cohort study of 1,016 patients on ICIs, PPI use was a strong risk factor for AKI lasting 48 hours or more after 2.5 months of follow-up (adjusted hazard ratio, 2.85; 95% confidence interval, 1.05-6.08; *P* = 0.007).^12^ Other medications associated with AIN such as nonsteroidal anti-inflammatory drugs or antibiotics may also contribute to ICI-induced AIN.^19^^,^^20^ It is hypothesized that ICIs induce loss of tolerance to these potential haptens. In a large multicenter cohort, patients who developed ICI-induced AKI who were receiving AIN-associated medications concurrently had a higher chance of AKI recovery, likely because these medications can be discontinued, facilitating full recovery.^19^ This demonstrates the importance of stopping other potential AIN-provoking medications to increase the chances of recovery in patients with ICI-induced AKI. Another important clue while evaluating the cause of AKI in a patient receiving ICIs is the occurrence of nonrenal immune-related adverse events.^19^ A patient concurrently or recently diagnosed with ICI-induced rash, colitis, myocarditis, or thyroiditis, for example, is more likely to also have an immune-related adverse event in another organ such as the kidney. Such patients have a propensity to develop activated T cells against autoantigens if the intensity threshold for an immune response against self tissue has already been reached. However, a concurrent immune-related adverse event is not a sensitive indicator of ICI-induced AKI; a large, multicenter study showed that extrarenal immune-related adverse events occurred in 43% of patients diagnosed with ICI-induced AKI.^19^ Finally, though the median onset of ICI-induced AKI is around 12 weeks after initiation of ICIs, it can occur at any time posttherapy, from as early as a few days after the first dose to >10 weeks after the last dose.^19^

It should be noted that ICIs are increasingly being combined with conventional chemotherapy, targeted antineoplastic therapy, novel immunotherapies, or radiation. Combination of immunotherapy and traditional chemotherapy, such as triple therapy for lung cancer that includes carboplatin, pembrolizumab (a programmed cell death-1 [PD-1] inhibitor), and pemetrexed, poses important diagnostic challenges, as all 3 of these drugs may lead to AKI. Understanding the chances of toxicity and the specific lesions associated with each agent and performing a detailed work-up, including a kidney biopsy as appropriate, are needed to differentiate between nephrotoxicity associated with chemotherapy and ICI-induced AKI.^21^ This is critical to ensure that potentially effective therapies are not unnecessarily discontinued. It is unknown if concurrent treatment with nephrotoxic chemotherapy agents increases the risk of ICI-induced AKI, and further studies are required in this rapidly-growing area of combination chemotherapy/ICI therapy.

### Patterns of kidney injury

AIN is not the only kidney manifestation of ICIs. While AIN is the most common pathology seen with ICIs, (affecting >90% of patients who underwent biopsy in a large multicenter series),^19^ pathologies affecting other compartments of the kidney have also been reported. Acute tubular necrosis, thrombotic microangiopathy, and multiple glomerular diseases have been reported.^6^^,^^11^^,^^12^^,^^22^ While the true incidence of these lesions is not known, we speculate that they likely cause less than 10% of all ICI-induced AKI. In a systematic review of 45 biopsy-proven cases of glomerular disease occurring in patients on ICIs, pauci-immune glomerulonephritis/vasculitis (27%), podocytopathies (20%), and C3 glomerulopathy (11%) were the most common.^22^ The median time to glomerular disease was 3 months after ICI initiation, which is similar to ICI-induced AIN. Interestingly, 40% of the patients with glomerular pathologies also had concomitant AIN. In patients with pauci-immune glomerulonephritis/vasculitis (n = 12), antineutrophil cytoplasmic antibody (ANCA) testing was negative in all but 2 cases, and none of the patients were on drugs typically associated with ANCA vasculitis such as hydralazine or minocycline. The authors hypothesize that immune hyperactivity and T-cell mediated activation of neutrophils may expose epitopes on the neutrophil surface, against which preformed ANCA may react. The ANCA negativity in these cases may be due to as yet unidentified epitopes (not proteinase-3 or myeloperoxidase) against which ANCA can act. The outcomes reported in patients with de novo ICI-induced glomerular diseases are poor; despite 98% receiving corticosteroid treatment and 73% experiencing complete or partial recovery of AKI, only 12% could be rechallenged with ICI, 19% progressed to end-stage kidney disease, and one-third died.^22^

ICIs may reactivate pre-existing autoimmune diseases.^23^ Two multicenter series reporting clinical outcomes in patients with pre-existing autoimmune disease (rheumatoid arthritis, lupus, and inflammatory bowel disease) who received ICIs, between 27%-50% of the patients experienced an exacerbation of their autoimmune disease.^24^ To our knowledge, only 1 case of an adverse kidney outcome has been described in a patient with pre-existing glomerulonephritis: a recurrence of primary membranous nephropathy.^25^ There is also a case report of a patient with pre-existing ANCA vasculitis who received PD-1 inhibitor therapy successfully without inducing a flare of vasculitis.^26^

In addition, distal renal tubular acidosis can be a manifestation of incipient or persistent AIN. In some cases, distal renal tubular acidosis can persist despite improvement of kidney function while tapering corticosteroid therapy.^27^^,^^28^ In these cases, analysis of kidney biopsies by immunofluorescent labeling with specific antibodies shows loss of intercalated cells expressing anion exchanger type 1 and B1 and α4 subunits of vacuolar-type H⁺-ATPase as compared to controls, elucidating in part the mechanism of autoimmunity.^28^

### ICI and kidney transplant

Solid organ transplant recipients are at increased risk of developing and dying from cancer in comparison to the general population.^29^^,^^30^ While ICIs are efficacious in a variety of malignancies, there are distinct challenges in kidney transplant recipients. ICIs activate the patient’s own immune system against the cancer, but off-target effects on the allograft can occur because PD-1 and programmed cell death-ligand 1 (PD-L1) receptors can be found in the allograft and its reactive T cells.^31^ Following ICI treatment, approximately 40% of kidney transplant recipients develop rejection with a median time from ICI initiation to rejection of 22-24 days.^32^^,^^33^ Factors associated with a lower risk of rejection include the use of mammalian target of rapamycin inhibitors and triple (as opposed to dual) agent immunosuppression.^32^^,^^34^ However, other important factors include transplant duration, history of donor-specific antibodies, or prior history of rejection. More studies are needed to determine how best to use these new cancer agents to improve outcomes in transplant recipients.

### Differences between ICI classes

Not all ICIs carry the same risk of kidney disease. Cytotoxic T lymphocyte antigen-4 inhibitors (ipilimumab) and PD-1 inhibitors (pembrolizumab, nivolumab, and cemiplimab) were the first approved ICI classes. Initially, pooled data from clinical trials showed that the frequency of kidney disease was similar among these 2 classes of ICIs; Cortazar et al^6^ showed that both classes had an ICI-induced AKI frequency of around 2%. Subsequent real-world data suggested that while the overall incidence was slightly higher (estimated to be 3%-5%), there was no data to indicate that one class was associated with fewer kidney-related adverse effects than the other. However, the combination of cytotoxic T lymphocyte antigen-4 and PD-1 inhibitors, used to induce a more durable response in cancers such as non–small cell lung cancer, melanoma, or renal cell carcinoma, carries a higher risk of ICI-induced AKI (∼5%) due to the blockade of 2 different checkpoint pathways.^6^^,^^10^, ^11^, ^12^ The most recently approved class of ICIs, PD-L1 inhibitors, includes atezolizumab, durvalumab, and avelumab, and they are now being widely used for multiple cancer types.^35^ Interestingly, the selective nature of this class of ICIs, which blocks PD-1/PD-L1 interaction but not PD-1/PD-L2 interaction, may provide the advantage of being less organ-toxic than other ICI classes, with trial data showing less pneumonitis reported with these drugs compared to PD-1 inhibitors and less colitis when compared to cytotoxic T lymphocyte antigen-4 inhibitors.^36^, ^37^, ^38^, ^39^ A retrospective cohort study of nearly 600 patients receiving PD-L1 inhibitors demonstrated <1% incidence of PD-L1-related AKI, which is a notably lower incidence than the 2%-5% incidence reported with other classes.^20^ This finding needs to be validated at other centers. With more checkpoint pathways being investigated as potential therapeutic targets,^40^^,^^41^ and each purported to have differential effects on the immune system, nephrologists will need to carefully determine kidney-specific effects of each ICI class. It should also be noted that ICIs have long half-lives (6-27 days) since they are primarily cleared by proteolytic degradation within the liver; kidney dysfunction does not meaningfully affect elimination.^42^ However, the half-lives differ by ICI agent, for example, atezolizumab (27 days) and avelumab (6 days). It is unclear if this plays a role in inducing ICI-induced AKI or if the half-life impacts the duration of corticosteroids needed to treat immune-related adverse events. Future research is needed in this area.

### Potential aids in clinical decision making beyond the standard of care

A kidney biopsy is the gold standard for diagnosing ICI-induced AIN or ICI-associated glomerular disease. However, current guidelines suggest empiric treatment of suspected ICI-induced AIN is acceptable if other causes have been ruled out and glomerular disease is not suspected.^43^^,^^44^ The decision on whether a kidney biopsy should be performed should be individualized with patient- and cancer-related factors taken into account (Box 1).^8^

Box 1 **Factors for and Against Performing a Kidney Biopsy in Cases of AKI After ICI**

**Table:** 

Favors Performing Kidney Biopsy to Conclusively Diagnose Etiology of ICI-Induced AKI	Favors Empiric Treatment of Presumed ICI-Induced AIN
• Grade 2 or 3 AKI • Lack of other concurrent immune-related adverse event at the time of AKI and no concomitant AIN-associated medications (PPI, NSAIDS, antibiotics) • Other potential etiologies that are equally likely and cannot be ruled out with history or laboratory testing along • Concurrently receiving other nephrotoxic antineoplastic therapies • Presence of proteinuria >1 g/day • Serologic abnormalities (such as positive ANCA, hypocomplementemia) • Low risk for biopsy procedure (BMI <30 kg/m^2^, no prior episodes of significant bleeding, no current coagulopathy, well controlled hypertension, not on antiplatelets or anticoagulants)	• Concurrently experiencing other nonrenal immune-related adverse events • Concurrently taking other AIN-associated medications (PPI, NSAIDS, antibiotics) • One or more risk factors for bleeding complications (BMI >30 kg/m^2^, prior intracranial or transfusion-requiring bleeding, uncontrolled hypertension with SBP >160 mm Hg despite antihypertensives, on antiplatelets or anticoagulants, patient with altered mental status, mechanical ventilation) • Solitary functioning kidney or multiple cysts in the kidney • Urgent need to treat with empiric steroids (AKI-requiring RRT) when kidney biopsy is not immediately feasible.

*Note:* The decision between confirming an ICI-induced AIN diagnosis through a kidney biopsy versus empiric immunosuppression should weigh the individual’s risk of procedural complications against the side effects of inadvertent steroids in the case of a misdiagnosis.Abbreviations: AIN, acute interstitial nephritis; AKI, acute kidney injury; ANCA: antineutrophil cytoplasmic antibody; BMI, body mass index; ICI, immune checkpoint inhibitor; NSAIDS, nonsteroidal anti-inflammatory drugs; PPI, proton pump inhibitor; RRT, renal replacement therapy; SBP, systolic blood pressure.

Currently, validated noninvasive markers for the diagnosis of ICI-induced AKI do not exist, and the use of many of the tools mentioned hereafter are limited to research studies. When biopsies are performed, additional stains to confirm ICI-induced AKI may be used to confirm association with ICIs; positive staining of tubular epithelial cells for PD-L1 has been shown to help differentiate PD-1-related AIN from other AIN.^45^ However, the clinical utility has not been validated, and larger studies are needed before the PD-L1 stain can be recommended for this purpose. Noninvasive tests in patients unable to undergo a kidney biopsy are limited. Positron emission tomography imaging showing increased ^18^F-fluorodeoxyglucose uptake in the renal cortex may be a valuable adjunct test to confirm ICI-induced AIN, especially in those with a baseline positron emission tomography scan for comparison.^46^^,^^47^ There have also been serum and urine biomarkers that have shown promise in small series of patients. A recent study by Isik et al^13^ found that patients with ICI-induced AKI have higher levels of serum C-reactive protein and urine retinol-binding protein/urine creatinine compared to patients with non–ICI-induced AKI. These biomarkers, when used in combination and in the right clinical context (other infectious and inflammatory causes being ruled out) may serve well to indicate the presence of ICI-induced AKI. On the contrary, when both biomarkers are within normal limits, the likelihood of an ICI-induced AKI is very low.^13^ In another study in lung cancer patients, neutrophil-to-lymphocyte and platelet-to-lymphocyte ratios were predictive of immune-related adverse events.^48^ Current research focusing on immunological markers specific to ICI-induced immune dysregulation, such as T-cell repertoire and profiling of gene expression may provide sophisticated ways to analyze and detect ICI-induced AIN and other immune-related adverse events.^49^^,^^50^ These and other novel biomarkers of AIN, such as urine tumor necrosis factor-α and interleukin-9,^51^ need further study and validation in larger, multicenter studies, as noninvasive markers of ICI-induced AIN are desperately needed.

## Management

### Impact of ICI-induced AKI on outcomes

While the occurrence of an immune-related adverse event such as AIN may threaten to derail effective cancer treatment, emerging evidence suggests that immune-related adverse events may be associated with improved overall survival.^52^ Immune-related adverse events may be a marker of a “desirable” immune response, with higher-grade immune-related adverse events predicting or acting as a marker for a higher immunotherapy-objective response rate and longer progression-free survival in cancers such as renal cell carcinoma and non–small cell lung cancer.^53^ It is unknown if specific immune-related adverse events such as AIN have the same association with mortality. The goal of management of ICI-induced AKI should be to prevent kidney failure while keeping in mind the importance of ongoing cancer care.

### Personalized approach to the management of ICI-induced AKI

Current guidelines for ICI-induced AKI are formulated by the Society for Immunotherapy of Cancer per Common Terminology Criteria for Adverse Events (CTCAE version 5.0) (Table 1).^54^ For persistent grade 2 (doubling of creatinine or higher), the current guidelines recommend discontinuing ICIs. They also recommend a corticosteroid taper that begins when creatinine improves to grade 1 or below. The guidelines from the National Comprehensive Cancer Network are similar and specify a starting dose and duration for the corticosteroid taper: 0.5-1 mg/kg/day for grade 2 and 1-2 mg/kg/day for grade 3 AKI with the dose being tapered over 4-6 weeks after creatinine decreases to less than or equal to grade 1; there is also an additional National Comprehensive Cancer Network recommendation to consider additional immunosuppression (cyclophosphamide, azathioprine, cyclosporine, infliximab or mycophenolate) if AKI does not improve to less than grade 2 within 1 week.^44^

**Table 1:** Comparison of Guidelines Based Recommendations for Management of ICI-Induced AKI

Management	NCCN	SITC	ASCO
Immunotherapy	G1 & G2: Hold; G3 & G4: Permanently discontinue	G1 & G2: Hold; G3 & G4: Permanently discontinue	G1 & G2: Hold; G3 & G4: Permanently discontinue
Kidney Biopsy	Consider for G3	No recommendation	Consider kidney biopsy if alternative causes cannot be ruled out
Corticosteroid Taper	G1: None; G2: 0.5-1 mg/kg/day; G3/G4: 1-2 mg/kg/day; Taper over 4-6 weeks once Cr <=G1; Monitor Cr weekly	Dose/schedule to be individualized and based on grade	G1: None; G2: 0.5-1 mg/kg/day; G3/G4 or no improvement or worsening in G2: 1-2 mg/kg/day; Taper over 4-6 weeks once Cr <=G1; Monitor Cr weekly
Other immunosuppression	Add immunosuppression (cyclophosphamide, mycophenolate, azathioprine, infliximab) if Cr> G2 after 1 week	No recommendation	Add immunosuppression(e.g. Mycophenolate) of worsening or no improvement in: 7 days (G2)/3-5 days (G3)/2-3 days (G4)

*Note:* Recommendations by major societies or expert working groups on the management of checkpoint inhibitor related acute kidney injury.^43^^,^^44^^,^^63^ Grading of renal immune-related adverse event is based on CTCAE (Common Terminology Criteria for Adverse Events) v5.0 criteria and is defined by elevation of creatinine above baseline.^54^ Mild (G1): 1.5-2× baseline Cr or 0.3 mg/dL elevation above baseline; Moderate (G2): 2-3× baseline Cr; Severe (G3): >3× baseline Cr; Life-threatening (G4): >6× baseline Cr or dialysis indicated.

Abbreviations: ASCO, American Society of Clinical Oncology; Cr, creatinine; NCCN, National Comprehensive Cancer Network; SITC, Society for Immunotherapy of Cancer.

**Figure 2:**
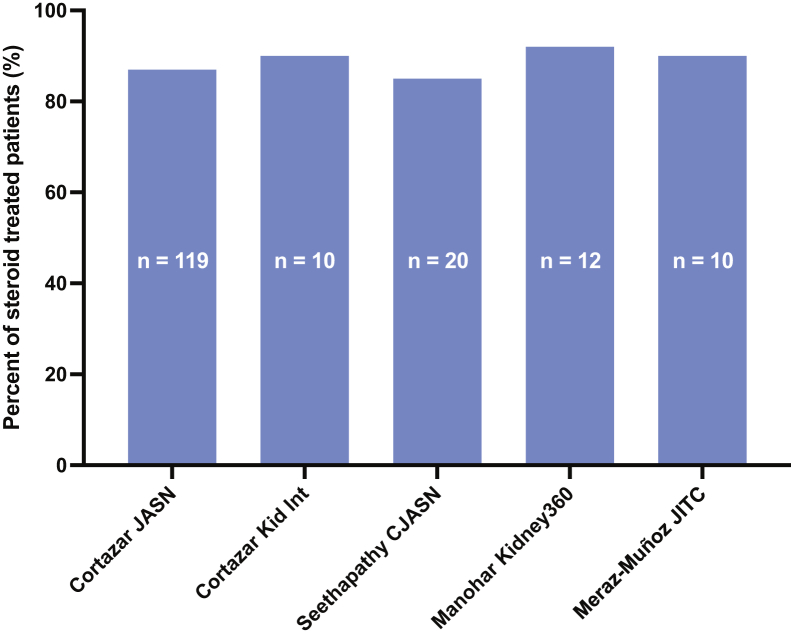
Percentage of steroid-treated patients with immune checkpoint inhibitors (ICI)-induced acute interstitial nephritis (AIN) that achieved full or partial remission. Studies with ≥10 patients with ICI-AIN treated with steroids were included. The typical steroid courses used in these studies include a starting dose of 40-100 mg/day (0.5-1 mg/kg/day) tapered over 4-12 weeks.^6^^,^^11^^,^^12^^,^^14^^,^^19^

Multiple series have shown that approximately 85% of patients with ICI-induced AKI who receive corticosteroids have full or partial remission (Fig 2). Cortazar et al^19^ studied 138 patients with ICI-related AKI; 119 were treated with corticosteroids (n = 119)–43% had partial and another 44% had a full recovery. The need for second-line immunosuppression was rare in this series. The difference in long-term kidney outcomes between patients who experience partial versus complete recovery is unknown, and this is an important area for future investigation. In the minority of patients who develop relapsing or persistent AKI, infliximab may be a possible treatment option. A series of 10 patients with relapsed ICI-induced AKI showed that 80% achieved a durable complete or partial remission after receiving infliximab.^55^ There is limited data with the use of other agents such as mycophenolate mofetil, and its use may not be ideal as it inhibits directly activated T and B cells. Further research is required before such agents are recommended.^56^^,^^57^ While AKI has not been shown to be associated with increased mortality, AKI nonrecovery has been shown to be a marker of increased mortality.^19^ Hence, AKI needs to be promptly evaluated and when detected, early glucocorticoid initiation is crucial. While deciding on a glucocorticoid course, it is important to consider their adverse effects and that patients may be at risk for cancer relapse. There is some controversy and concern that glucocorticoid treatment may diminish the tumor-directed T-cell response and lead to cancer progression.^58^^,^^59^

Steroid-minimizing strategies that use a lower duration of therapy than current guidelines may be appropriate, particularly in patients who are concurrently receiving another AIN-associated medication, such as PPIs, which can be discontinued at the same time corticosteroids are initiated. In an observational study, Lee et al^60^ showed that patients with ICI-induced AKI who were treated with 3 weeks of prednisone had excellent and equivalent kidney outcomes compared to historical controls who were treated with longer courses (4-6 weeks) of prednisone. In this series, patients receiving shortened courses of corticosteroids had withdrawal of other AIN-associated medications (PPI, nonsteroidal anti-inflammatory drugs, and antibiotics). It should be noted that steroid-minimizing strategies require careful patient selection as shown in Box 2. Furthermore, these results need to be validated in larger, prospective studies.

Box 2 **Factors Influencing Steroid Course and the Decision to Rechallenge With ICIs**

**Table:** 

Factors Favoring Quick Steroid Taper and ICI Rechallenge	Factors Favoring Longer Steroid Course and Avoiding ICI Rechallenge
• No other severe immune-related adverse events (myocarditis, myositis, pneumonitis, hepatitis, neurologic immune-related adverse events) • AKI that recovers quickly with corticosteroids (begins improving in <1 week) • Other AIN-associated medications that can be discontinued (PPI, NSAIDs, antibiotics, etc.) • Biopsy or clinical features of AIN (as opposed to other immune-mediated glomerular diseases) • Newly starting therapy (patient has likely not yet derived possible anticancer benefit) • Melanoma and other ICI-sensitive tumors • ICI used has a short half-life	• AKI slowly recovering with <25% change in creatinine by 5-7 days • Evidence of ICI-associated glomerular disease on biopsy or nephrotic-range proteinuria • No concomitant AIN triggering medications • Other life-threatening immune-related adverse events (myocarditis, myositis, pneumonitis, hepatitis, or neurologic) • Cancers that are not particularly sensitive to ICIs • Longer duration on therapy and stable cancer (suggesting that whatever benefits are to be gained have already been realized and cancer is likely to be stable off ICIs)

*Note:* Steroid-responsive AIN in the absence of extrarenal immune-related adverse events allows for a quick steroid taper and ICI rechallenge.Abbreviations: AIN, acute interstitial nephritis; AKI, acute kidney injury; ICI, immune checkpoint inhibitor; NSAID, nonsteroidal anti-inflammatory drug; PPI, proton pump inhibitor.

Finally, it is unclear if patients on ICIs with short half-lives and reduced duration of action such as avelumab may have better kidney outcomes with shorter steroid tapers when compared to those on drugs with longer half-lives (eg, atezolizumab, pembrolizumab), where the duration of checkpoint inhibition may last much longer.

### Rechallenging patients with ICI-induced AKI

In ICI patients experiencing high-grade AKI, the ICI is typically interrupted until AKI resolves, and sometimes permanently discontinued. In the multicenter study by Cortazar et al,^19^ only 22% of 138 patients with stage 2 or 3 AKI were rechallenged. Recurrent ICI-induced AKI occurred in only 23% of rechallenged patients; of those with recurrent AKI, all but one responded to a second course of glucocorticoids. Other studies show a recurrence range of 8%-40% after rechallenge.^10^, ^11^, ^12^ The decision to rechallenge with ICI after an episode of ICI-induced AKI depends on patient- and cancer-related factors (Box 2). It should also be noted that while the incidence of recurrent ICI-induced AKI is relatively low, patients with ICI-induced AKI who are rechallenged with ICI may also be at risk of developing other immune-related adverse events, such as colitis, hepatitis, myocarditis, or pneumonitis, some of which could be life-threatening. In a series of 30 patients with ICI-induced AKI, 4 out of 17 who were rechallenged developed a severe extrarenal immune-related adverse event. Many oncologists choose to continue low-dose prednisone (5-10 mg/day) when attempting an ICI rechallenge; however, currently, there is no data to support such use. Further research is needed to identify the specific risk factors and biomarkers for recurrent AKI or the development of new immune-related adverse events on ICI rechallenge.

### Long-term effects of ICIs on kidney function

The long-term effects of ICIs on kidney function are not well known. It is well known that AKI is a risk factor for chronic kidney disease and progression to end-stage kidney disease. In a recent study of >2,500 patients treated with ICIs who survived at least 1 year, rapid estimated glomerular filtration rate decline (>3 mL/min/1.73 m^2^ per year) was common and the incidence of new-onset chronic kidney disease or significant (>30%) estimated glomerular filtration rate decline sustained for >90 days occurred in 20% of survivors who lived at least 5 years.^61^ While the exact reasons are unclear, multiple mechanisms are possible. Patients with overt ICI-induced AKI may fail to fully recover from ICI-induced AKI despite steroid treatment, immune activation caused by long-term exposure to ICIs may accelerate the progression of pre-existing kidney disease, ICIs may lead to subclinical interstitial nephritis, and finally, recurrent AKI episodes may contribute to accelerated chronic kidney disease. Future research will be needed to define the long-term kidney risks among survivors and to understand the mechanisms of chronic kidney disease in patients receiving ICIs.

## Conclusion and Future Directions

Management of ICI nephrotoxicity is multipronged and should place emphasis on cancer survival and the quality of life of patients. Future research should focus on the identification of sensitive and specific markers that can diagnose and predict ICI-induced AIN, the development of personalized safe and effective immunosuppression protocols, as well as strategies for ICI rechallenge and management of recurrent AKI. Further research is needed to determine the mechanisms of ICI-induced glomerular diseases and determine the best strategies for managing these rarer kidney complications. The long-term risks of ICIs on kidney function need to be better understood as the number of patients being treated with ICIs is rapidly growing; ICIs are now estimated to be indicated in 36% of patients with cancer.^62^ As new ICIs are introduced, nephrologists and researchers should use current understanding as a framework for exploring the differences in the incidence, risk factors, and outcomes with these drugs. Nephrologists should also play an active role in the development of oncologic guidelines for the management of ICI-induced AKI and contribute to the understanding of ICI-induced immune-related adverse events as a whole. This may lead to the development of biomarkers or treatment strategies that could be applied more broadly to patients with other forms of AIN.
